# Mirror-image streptavidin with specific binding to L-biotin, the unnatural enantiomer

**DOI:** 10.1038/s41598-022-13763-4

**Published:** 2022-06-10

**Authors:** Masatoshi Suganuma, Takuya Kubo, Kengo Ishiki, Kota Tanaka, Kouzou Suto, Daisuke Ejima, Masahiro Toyota, Kouhei Tsumoto, Toshiyuki Sato, Youichi Nishikawa

**Affiliations:** 1 Central Research Laboratories, Sysmex Corporation, Kawasaki, Kanagawa 210-0821 Japan; 2 Central Research Laboratories, Sysmex Corporation, Kobe, 651-2271 Japan; 3 Bio-Diagnostic Reagent Technology Center, Sysmex Corporation, Kobe, 651-2271 Japan; 4 Bio-Diagnostic Reagent Technology Center, Sysmex Corporation, Sayama, 350-1332 Japan; 5 Department of Chemistry, Graduate School of Science, Osaka Prefecture University, Sakai, 599-8231 Japan; 6 Institute of Medical Sciences, The University of Tokyo, Tokyo, 108-8639 Japan; 7 Department of Bioengineering, School of Engineering, The University of Tokyo, Tokyo, 113-8656 Japan

**Keywords:** Biotechnology, Chemical biology

## Abstract

The streptavidin–biotin system is known to have a very high affinity and specificity and is widely used in biochemical immunoassays and diagnostics. However, this method is affected by endogenous D-biotin in serum sample measurements (biotin interference). While several efforts using alternative high-affinity binding systems (e.g., genetically modified streptavidin and biotin derivatives) have been attempted, these efforts have all led to reduction in affinity. To solve this interference issue, the enantiomer of streptavidin was synthesized, which enabled specific binding to L-biotin. We successfully obtained a functional streptavidin molecule by peptide synthesis using D-amino acids and an in vitro folding technique. Several characterizations, including size exclusion chromatography (SEC), circular dichroism spectra (CD), and heat denaturation experiments collectively confirmed the higher-order enantiomer of natural streptavidin had been formed with comparable stability to the natural protein. L-biotin specific binding of this novel molecule enabled us to avoid biotin interference in affinity measurements using the Biacore system and enzyme-linked immunosorbent assay (ELISA). We propose the enantiomer of streptavidin as a potential candidate to replace the natural streptavidin–biotin system, even for in vivo use.

## Introduction

Avidin and streptavidin are known to bind to natural biotin with high affinity and specificity^1,2^. Core streptavidin^3,4^, which consists of the core region of natural streptavidin, has particularly high stability against thermal and chemical denaturation, as well as protease degradation, and has therefore been used for various purposes in the life sciences field^5^. Its most widely used application is for highly sensitive quantification, exploiting the molecule’s high affinity for D-biotin for biological analysis. Numerous immunoassay systems using the streptavidin–biotin reaction for signal amplification or bound/free (BF) separation have been proposed^6,7^. However, the current limitation to the use of the streptavidin–biotin reaction is that endogenous biotin from human samples binds to streptavidin used in these biological assays, phenomena referred to as “biotin interference”, which may lead to false negative or false positive results depending on the assay. The unusually high concentrations of endogenous biotin in human samples are results of excess intake of foods or supplements containing D-biotin. Regulatory authorities, including the US Food and Drug Administration (FDA), required reliable action to be taken by diagnostic companies to solve this misinterpretation of results caused by biotin interference in clinical assays^8^. Streptavidin variants with low affinity for D-biotin and high affinity for biotin derivatives have been proposed^9–11^. However, the affinity for these biotin derivatives have so far proved to be inferior to the affinity of natural streptavidin for D-biotin, showing insufficient applicability of these newly proposed biotin derivatives for avoiding biotin interference.

In this study, we chose another approach to solve the biotin interference issue. Chirality works as an important factor in determining biomolecular interactions in broad range of scales, from small molecules to nanoparticles^12^. The key to our approach is to create a protein composed of D-amino acids, instead of L-amino acids, creating the enantiomer of the natural protein. This approach would provide us the same interaction of a set of natural partner molecules because no structural modifications were made except for the optical isomer relationship. We expect that this approach would provide high selectivity to L-biotin, instead of D-biotin, thereby avoiding biotin interference in the biological samples. This assumption is based on the strict molecular recognition formerly confirmed by the X-ray crystallography of natural streptavidin and the D-biotin complex.

Several critical studies have reported the application of D-amino acids for enzymes. Such studies involved HIV protease and DNA ligase, composed of D-amino acids, which were prepared and characterized for these functions^13–15^. Discovery stage studies using therapeutic peptides have shown the potential applicability of reciprocal chiral specificity; L-peptides act as antagonists against the D-target protein, while the D-peptide strictly antagonizes the natural L-target form of the protein^16^. Functional D-peptides have been discovered using mirror-image phage display techniques^17,18^ and have been developed for several applications^19,20^. In contrast, synthetic D-amino acid peptide mimics of the HIV-1 gp41 N-terminal sequence were shown to associate with the wild type gp41fusion peptide, indicating that the chirality of the peptide was not necessarily required for the interaction^21^. Peptide fragments composed of D-amino acids of Abeta_1-42_ inhibited natural Abeta_1-42_ fibril formation, indicating peptide/peptide interactions without critical chiral specificity^22^. Molecular chaperones can fold both the L- and D-substrate proteins^14^. Latter cases with loose chiral specificity might indicate room for chiral recognition on proteins or peptides. We should carefully evaluate the rigidity of the chiral specificity in each molecular system.

In our study, we expected strict chiral-specific recognition in the streptavidin–biotin system based on the accurate molecular packing and ultra-high affinity observed for natural streptavidin and D-biotin. We synthesized core streptavidin peptide consisting of D-amino acids, in place of L-amino acids, using peptide synthesis, which gave rise to a functional form (D-core streptavidin) which displayed a native high-order structure. The high-order structure and thermal stability of the obtained D-core streptavidin were compared with those of the natural core streptavidin. Furthermore, the functions of D-core streptavidin, in particular the L-biotin binding affinity and suppression of D-biotin interference in the immunoassay for biological samples, were evaluated. Finally, the applicability of this novel interaction using the D-core streptavidin and L-biotin are discussed.

## Results and discussion

### Synthesis

The core streptavidin sequence is known to be 127 amino acid residues, as shown in Table 1. The core streptavidin peptide composed of D-amino acids and achiral glycine was synthesized using GlyTech Inc. peptide synthesis service. The entire core streptavidin sequence was divided into five segments and each segment was prepared by Fmoc solid-phase synthesis with D-amino acids and glycine. The total amount of synthesized peptide was 79.5 mg. As for immunoassay evaluation, the core streptavidin sequence was divided into 2 segments to accelerate its synthetic process. The full-length D-amino acid polypeptide was obtained by linking the segments using the general ligation method. The sequence corresponded to residues 37–163 in the amino acid sequence of natural streptavidin. The Electrospray Ionization Mass Spectroscopy (ESI–MS) spectrum and High performance liquid chromatography (HPLC) of the peptide synthesized from the five segments was shown in Figure S2 and S3.

**Table 1:** Core streptavidin amino acid sequence.

AEAGITGTWYNQLGSTFIVTAGADGALTGTYESAVGNAESRYVLTGRYDSAPATDGSGTALGWTVAWKNNYRNAHSATTWSGQYVGGAEARINTQWLLTSGTTEANAWKSTLVGHDTFTKVKPSAAS	

**Figure 1:**
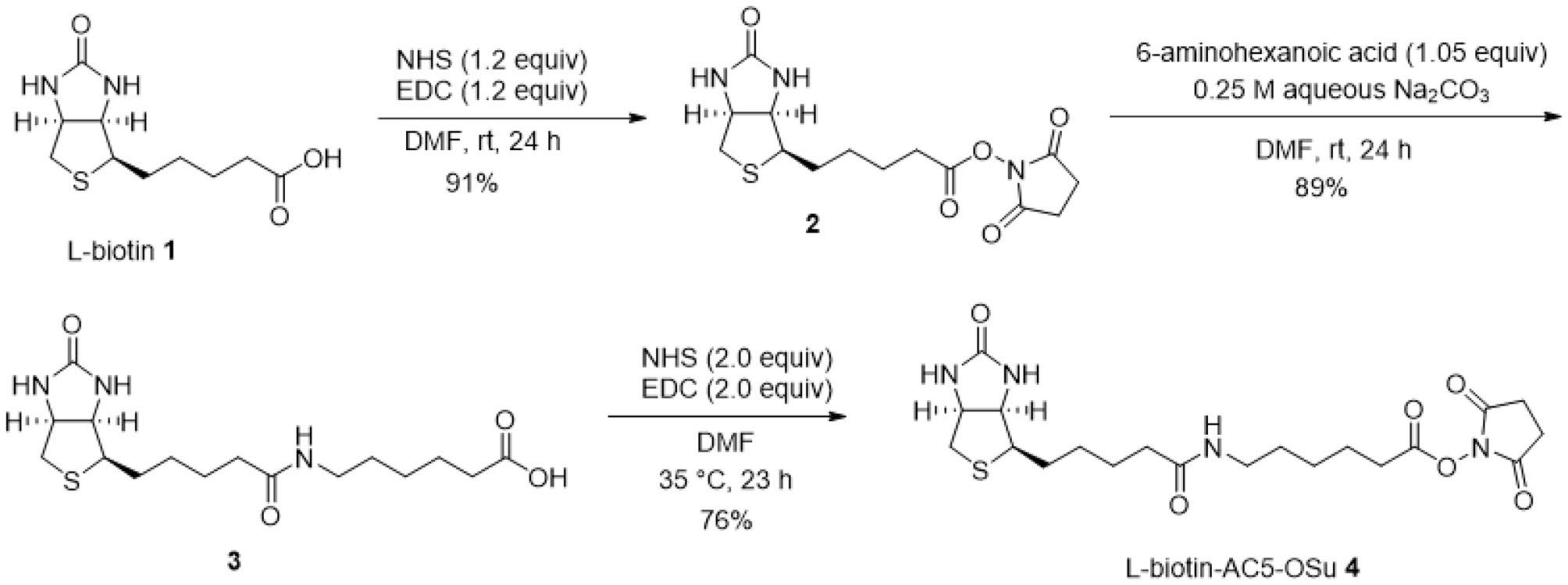
Synthesis of L-biotin-AC5-OSu. PerkinElmer, ChemDraw professional version 17.1.0.105(19) (https://perkinelmerinformatics.com/products/research/chemdraw/).

We also prepared L-biotin and its derivatives. L-biotin was obtained by performing optical resolution from racemic biotin synthesized according to a previous report^23^. Furthermore, 2,5-dioxopyrrolidin-1-yl 6-(5-((3a*R*,4*R*,6a*S*)-2-oxohexahydro-1*H*-thieno[3,4-*d*]imidazol-4-yl)pentanamido)hexanoate (L-biotin-AC5-OSu) was synthesized from L-biotin as a material for L-biotin modification to the ε-amino group of proteins. A synthetic route of L-biotin-AC5-OSu is shown in Fig. 1. The ^1^H Nuclear Magnetic Resonance (NMR) spectrum of L-biotin-AC5-OSu was sown in Figure S4.

### Folding of D-core streptavidin and natural core streptavidin

Tetrameric high-order structures of D-core streptavidin and the natural core streptavidin were obtained by well-known dilution folding^24^ with some modifications (Table 2). Representatively, D-core streptavidin peptide (28.8 mg; synthesized) and natural core streptavidin powder (10.1 mg; Roche diagnostics) were solubilized and denatured in 6 M guanidine hydrochloride at a protein concentration of approximately 20 mg/ml for 45 min at 85 °C, and then diluted sixfold with 80 mM Tris–HCl buffer to 1 M guanidine hydrochloride and kept for 30 min at room temperature. Small amounts of insoluble aggregates were observed during this step. Diluted solutions were then concentrated sixfold and applied to the second 100-fold dilution with 80 mM Tris–HCl buffer, pH 8.0. Diluted solutions were then concentrated 200-fold by centrifugation at 4 °C. Obtained supernatants were kept at 4 °C for 12 h to remove non-native by-products as insoluble aggregates. Finally, protein mixtures were centrifuged at 4 °C to yield natively folded core streptavidin proteins with no aggregates. Folded D-core streptavidin and natural core streptavidin were obtained with recovery yields of 25% and 50%, respectively. The suppression of the recovery yield for D-core streptavidin (25%) may be due to less pure folding materials compared to the purchased recombinant protein, the natural core streptavidin (Roche Diagnostics). Each streptavidin sample was subjected to buffer exchange to 20 mM phosphate buffer (pH 8.0) and then used in the following experiments. The representative protein recoveries during denaturation and dilution folding are summarized in Table 2.

**Table 2:** Folding recovery of each core-streptavidin.

Step	Dilution fold (from Step 1)	Final solution condition of each step	Temperature (℃)	Duration (hour)	D-core streptavidin (mg)	Natural core streptavidin (mg)
1	1	80 mM Tris–HCl, 1 mM EDTA, 6 M guanidine hydrochloride, pH 8.0	85	0.75	28.8*^1^	10.1*^1^
2	6	80 mM Tris–HCl, 1 mM EDTA, 1 M guanidine hydrochloride, pH 8.0	25	0.50	(–)*^2^	(–)*^2^
3	600	80 mM Tris–HCl, 1 mM EDTA, 10 mM guanidine hydrochloride, pH 8.0	4	18	(–)*^2^	(–)*^2^
4	3	(200-fold concentration in the same buffer above)	4	12*^3^	7.2*^1^	5.1*^1^

*1: quantitated by UV absorption at 280 nm.

*2: not determined.

*3: duration time after the final concentration.

### Monitoring of tetrameric structure formation

**Figure 2:**
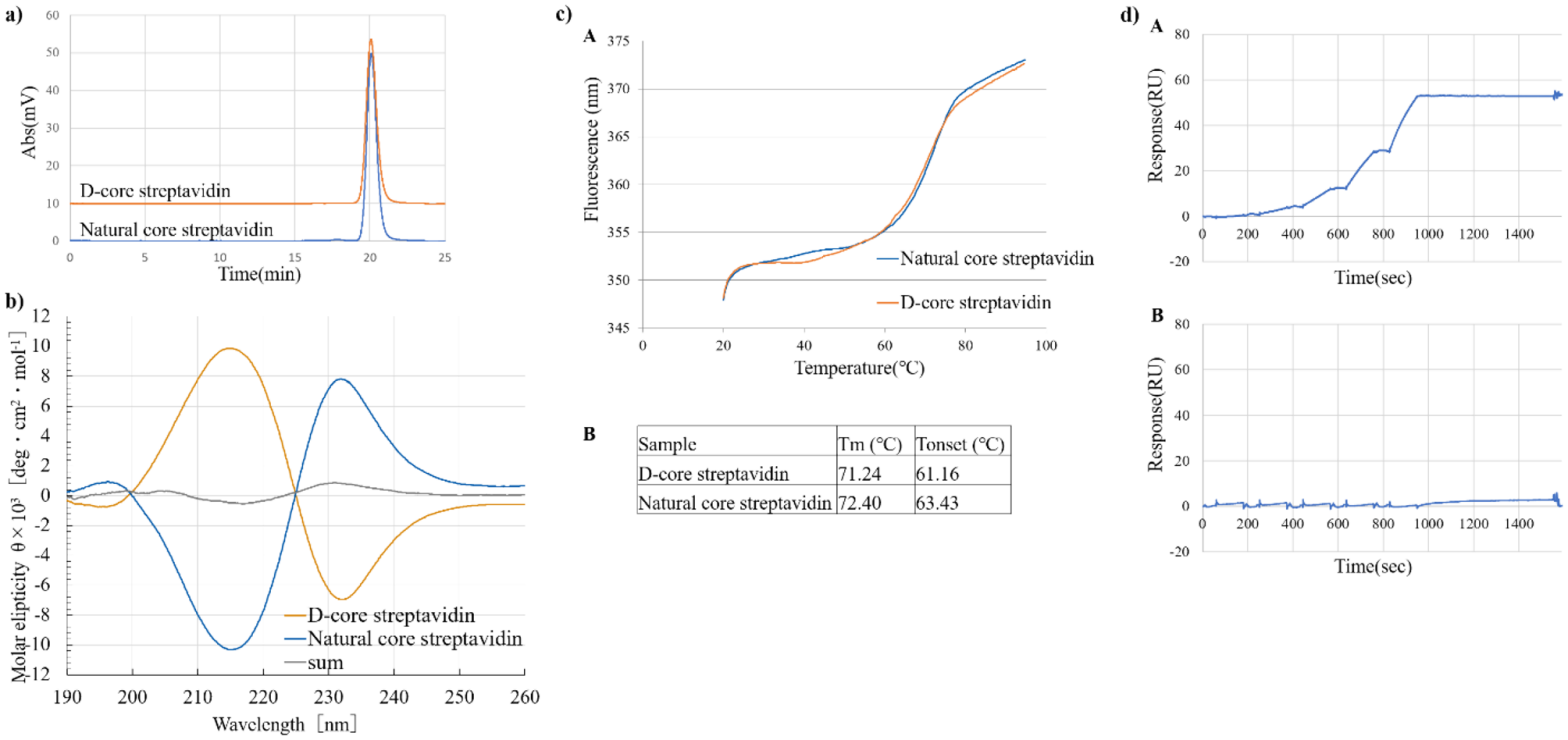
(**a**) SEC chart for core streptavidin. The horizontal axis shows the retention time (min), and the vertical axis shows the signal strength representing absorption at 280 nm. (**b**) CD spectra of folded D- and natural core streptavidin molecules (190–260 nm). sum: sum of CD spectra signals of D- and natural core streptavidin. (**c**) DSF curves of folded core streptavidin molecules A: DSF curves of folded D-core and natural core streptavidin molecules at concentrations of 0.5 mg/ml, B: Melting temperature (T_m_) and onset temperature (T_onset_) of folded natural core/D-core streptavidin. The concentrations of D-core streptavidin samples as analytes were 0.05, 0.15, 0.45, 1.35, 4.05 nM. (**d**) Biacore sensorgram for D-core streptavidin A: ligand, L-biotin; B: ligand, D-biotin.

The tetrameric structures of folded D-core streptavidin and natural core streptavidin were confirmed by analytical SEC (Fig. 2a). Both folded streptavidin enantiomers in 20 mM phosphate buffer showed symmetrical peak shapes with retention times comparable to that of the active streptavidin reagent (Figure S5). No other peaks with longer retention times were observed for either streptavidin, therefore only the tetramer was present. Peak areas of both streptavidins accounted for protein concentrations calculated by Ultraviolet (UV) absorbance, meaning there were no aggregates or unfolded forms that could not be eluted from the SEC column. After storage under refrigerated temperature or repetitive freeze–thaw, both streptavidin enantiomers showed unchanged SEC peak shapes (data not shown). These observations collectively suggest that our two-step folding was successful in the formation of a natively folded tetramer structure.

### Tertiary structures of streptavidin proteins

The higher-order structures of folded D-core streptavidin and its natural enantiomer, L-core streptavidin, in phosphate buffer were compared using circular dichroism spectra (CD spectra). CD spectra of natural core streptavidin have been reported^25^ with a negative ellipticity peak at 216 nm (derived from the β-sheet structures) and a positive ellipticity peak at 232 nm (presumably derived from the β-turn structures). In our studies, CD spectra (wavelength, 190–260 nm) of folded D-core streptavidin and the natural, L-core streptavidin are shown in Fig. 2b. These spectra showed characteristic ellipticities at 216 and 232 nm as seen in literature^25^), but with axial symmetry. It is well known that the CD spectra of compounds with optical isomerism show axial symmetry, especially in the case of low molecular weight chemicals with simple structures. However, it is not trivial whether a mirror image of the CD spectra is always the case with protein pairs composed of D- and L-amino acids, respectively. In proteins and peptides, for example, additional CD spectra at around 230 to 250 nm, which are derived from the side chains of aromatic amino acids, might overlap with the CD spectra of the main chains^26^. These situations might break the CD axial symmetries of L- and D-amino acid protein pairs. For example, DNA ligases composed of L- and D-amino acids, could not show accurate mirror images of CD spectra (axial symmetries) at around 230 to 250 nm^15^, where the influence of the aromatic amino acid side chains might occur. In contrast, in our study, obvious enantiomeric characteristics were observed between the CD spectra of the D-core streptavidin and the natural core streptavidin. The positive and negative ellipticities were clearly reversed at 216 nm and 232 nm, suggesting that numerous β-turn structures in D-core streptavidin have intense negative ellipticities at approximately 232 nm. Even if ellipticities derived from the aromatic side chains of D-core streptavidin overlap with ellipticities from its main chains, these effects would be minor. As for natural core streptavidin, CD spectra would be reversed compared to D-core streptavidin. As a result, we considered that mirror-image, higher-order structures of these two core streptavidin molecules showed substantial mirror image CD spectra. In addition, axial symmetries were observed even in the near-ultraviolet region of 250–300 nm (Figure S6). These results strongly suggest a mirror image of the secondary and tertiary structures of the D- and natural core streptavidin molecules.

### Thermal stabilities of streptavidin proteins

Thermal stability studies were performed using differential scanning fluorimetry (DSF). Heat denaturation curves and heat denaturation midpoint temperature (T_m_) of D-core streptavidin and natural core-streptavidin are shown in Fig. 2c. These two heat denaturation curves seemed to be quite similar and were estimated to have substantially comparable Tm values. The similar thermal stabilities observed here support the mirror image structures of the D-core and the natural core streptavidin molecules shown by CD spectra.

### Affinity and specificity of streptavidin proteins to biotin by kinetic and equilibrium analysis

Affinity analyses by Biacore were performed for folded core streptavidin molecules to determine their affinities with L- and D-biotin. We used D- and L-biotin-labeled bovine serum albumin (BSA) as ligands and D-core and natural core streptavidin as analytes. BSA was labelled by a commercially available D-biotin-AC5-OSu and above L-biotin-AC5-OSu. We first evaluated the appropriate BSA labelling conditions with biotin to obtain almost 1:1 binding between one streptavidin tetramer and one biotin on the surface of BSA. It was finally found that about 1.7 biotin molecules should react with one BSA molecule to avoid the avidity effect on binding. Sensorgrams of D-core streptavidin with L-/D-biotin are shown in Fig. 2d. While D-core streptavidin expectedly bound to L-biotin with sufficiently high affinity, no D-biotin binding was seen on the sensorgram. The equilibrium dissociation constants, K_D_s, of D-core streptavidin and natural core streptavidin reagent (11,520,679, Roche Diagnostics) to each ligand biotin were calculated by fitting curve analysis as 6.29 × 10^–14^ M and 6.85 × 10^–14^ M, respectively (Figure S7). Both streptavidin enantiomers might exhibit tetravalent binding sites, the K_D_ values were above the higher limit of affinity evaluation by Biacore kinetic analysis. Therefore, we in turn compared those K_D_ values by equilibrium (steady state) analysis and obtained 2.82 × 10^–9^ M for D-core streptavidin and 3.37 × 10^–9^ M for natural core streptavidin (Figure S8). In this study, the binding affinity observed between D-core streptavidin and L-biotin was comparable to that between natural core streptavidin and D-biotin. Importantly, the fact that D-core streptavidin did not show any binding affinity for D-biotin supported our hypothesis that stringent molecular recognition between streptavidin and biotin can exploit chiral specificity. Namely, D-core streptavidin proved to have a mirror image property in biotin binding.

### Evaluation of biotin interference on D-core streptavidin in immunoassay

**Figure 3:**
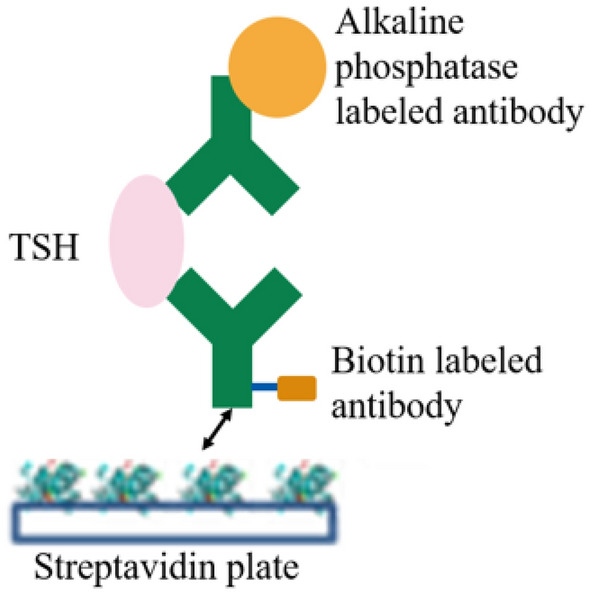
The architecture of the streptavidin–biotin sandwich CLEIA for TSH. Streptavidin images: BIOVIA, Dassault Systèmes, Discovery Studio Visualizer version 19.1(https://www.3ds.com/products-services/biovia/products).

**Figure 4:**
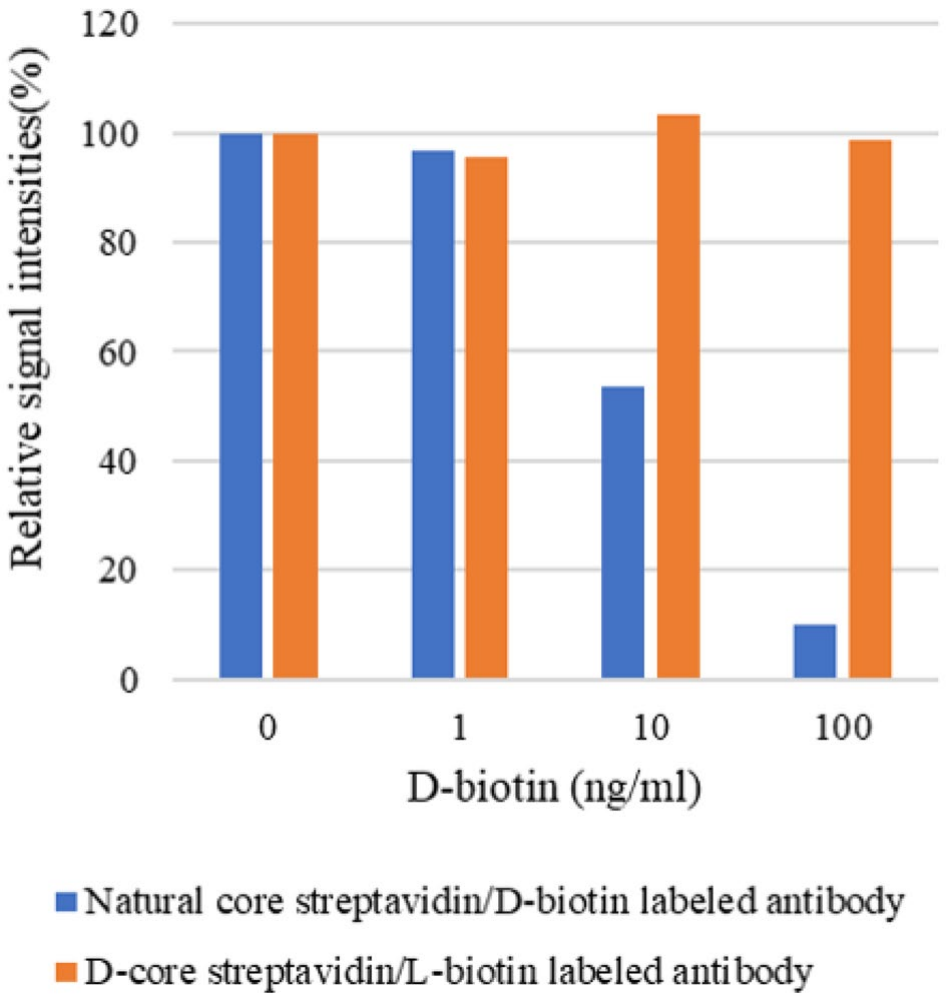
The effects of D-biotin added in the specimens for immunoassay. Horizontal axis: D-biotin concentration added in TSH specimens. Vertical axis: Relative signal intensities with various D-biotin concentrations added (D-biotin concentration: 0 nM, 100%). All values in the figure are expressed as mean from duplicated analysis.

The streptavidin–biotin reaction is a commonly used system in immunoassays^6,7^. We set up a streptavidin–biotin sandwich chemical luminescent enzyme immunoassay (CLEIA) system for thyroid stimulating hormone (TSH) to evaluate our D-core streptavidin capabilities. L-biotin labeled anti-TSH antibodies were prepared to capture TSH as the target antigen. D-core streptavidin reagents, as binding partners to biotin labeled anti-TSH antibodies, were immobilized in a 96-well plate for the immunosorbent assay. ALP-labeled anti-TSH antibodies were used for the sandwich detection. Various concentrations of D-biotin were added to TSH antigen specimens at a final concentration of 1–100 nM to observe the interference on its valid chemical luminescent signal. The architecture of the assay system is shown in Fig. 3. The results of signal interference are shown in Fig. 4. In the natural core streptavidin (reagent, 11520679, Roche Diagnostics)/D-biotin system, the signal decreased depending on the amount of D-biotin added, showing similar signal interferences seen in clinical diagnostics. However, no significant changes (signal decreases) in the D-core streptavidin/L-biotin system were observed, regardless of the amount of D-biotin added (± < 5%). We found that a strict molecular recognition system in streptavidin and biotin enabled us to avoid biotin interference through chiral specificity.

## Conclusion

The physicochemical properties shown by the SEC and CD spectra suggested that D-core streptavidin had a tetrameric and natively folded structure and was the enantiomer of a natural core streptavidin. In addition, the thermal stability shown by the DSF analysis supported this conclusion. We observed a very high binding affinity and specificity to L-biotin for D-core streptavidin. We also showed our D-core streptavidin together with L-biotin would be a solution to the issue of biotin interference, awaited in clinical diagnostics to be solved. We expect that our D-core streptavidin would have broader applicability, ranging from in vitro biochemical as-says to in vivo diagnostics, without endogenous biotin interference.

## Methods

### Synthesis of L-biotin derivatives

#### General

Unless otherwise noted, all reactions were performed in heat gun dried glassware, sealed with a rubber septum under an atmosphere of nitrogen or argon. Materials were obtained from suppliers and used without further purification. Flash column chromatography was carried out using Cica Silica Gel 60 N (spherical/40–50 μm). Reactions and chromatography fractions were analyzed employing precoated silica gel 60 F_254_ plates (Merck). Compounds were visualized using an ultraviolet lamp (254 nm) and/or by staining with *p*-anisaldehyde (in EtOH), potassium permanganate (in water containing NaOH and K_2_CO_3_) or ammonium molybdate (in 10% H_2_SO_4_). ^1^H NMR spectra were recorded on a Varian 400 MR (400 MHz) spectrometer and chemical shifts were reported relative to residual dimethyl sulfoxide (DMSO) (2.50 ppm).

#### L-biotin (1)

Racemic biotin was synthesized according to a previous report^23^ Optical resolution by high-performance liquid chromatography (HPLC) was conducted using an HPLC system (LC-20AD pump and SPD-20A detector, Shimadzu Co.) equipped with a CHIRALPAK IG column (Daicel Co., 4.6 mm (ID) × 250 mm, 5 mm). Solute elution was performed at a wavelength of 205 nm. Chromatographic conditions: column temperature, 40 °C; eluent, methanol/acetic acid = 100/0.1 (v/v); flow rate, 1.0 mL/min. [α]_D_^26^ = − 86.2° (*c* = 1.78, 0.1 M aqueous NaOH). [lit.^27^ D-biotin: [α]_D_^22^ =  + 91° (*c* = 1.0, 0.1 M aqueous NaOH)].

#### 2,5-Dioxopyrrolidin-1-yl 5-((3aR,4R,6aS)-2-oxohexahydro-1H-thieno[3,4-d]imidazol-4-yl)pentaneate (2)

To a stirred solution of L-biotin (**1**) (123 mg, 0.503 mmol) and *N-*hydroxysuccinimide (NHS) (69.4 mg, 0.603 mmol) in *N,N*-dimethylformamide (DMF) (3.40 mL) was added 1-(3-dimethylaminopropyl)-3-ethylcarbodiimide hydrochloride (EDC) (116 mg, 0.603 mmol) at room temperature. The reaction mixture was stirred at room temperature for 24 h. The solvent was evaporated in vacuo to give a pale brown syrup. The crude was recrystallized from ethanol/acetic acid/H_2_O (95:1:4 v/v) to afford **2** (166 mg, 91%) as a white solid.

#### 6-(5-((3aR,4R,6aS)-2-Oxohexahydro-1H-thieno[3,4-d]imidazol-4-yl)pentanamido)hexanoic acid (3)

To a stirred solution of **2** (166 mg, 0.458 mmol) in DMF (2.60 mL) were added 6-aminohexanoic acid (63.0 mg, 0.481 mmol) and 0.25 M aqueous Na_2_CO_3_ solution (1.00 mL) at room temperature. The reaction mixture was stirred at room temperature for 24 h. The solvent was evaporated in vacuo. The resulting white solid was dissolved in water. The aqueous layer was acidified with 4 M HCl solution at 0 °C. The precipitate was collected by filtration to afford **3** (159 mg, 89%) as a white solid. This material was used in the following reaction without purification.

#### 2,5-Dioxopyrrolidin-1-yl 6-(5-((3aR,4R,6aS)-2-oxohexahydro-1H-thieno[3,4-d]imidazol-4-yl)-pentanamido)hexanoate (L-biotin-AC5-OSu) (4)

To a stirred solution of **3** (159 mg, 0.408 mmol) and NHS (93.9 mg, 0.816 mmol) in DMF (7.60 mL) was added EDC (157 mg, 0.816 mmol) at room temperature. The reaction mixture was stirred at 35 ˚C for 23 h. The solvent was evaporated in vacuo to give a pale brown syrup. The crude product was purified by flash column chromatography with CH_2_Cl_2_/MeOH (8:1 v/v) to afford **4** (141 mg, 76%) as a white solid. ^1^H NMR (400 MHz, DMSO-d_6_) δ 1.22–1.65 (m, 12H), 2.04 (t, *J* = 7.2 Hz, 2H), 2.57 (d, *J* = 12.8 Hz, 1H), 2.65 (t, *J* = 7.2 Hz, 2H), 2.81 (s, 4H), 2.78–2.85 (m, 1H), 2.98–3.04 (m, 2H), 3.07–3.12 (m, 1H), 4.10–4.14 (m, 1H), 4.28–4.32 (m, 1H), 6.29 (s, 1H), 6.40 (s, 1H), 7.75 (t, *J* = 1.8 Hz, 1H). ESI–MS *m/z* 455.1967 [M + H]^+^ (*calcd for* C_20_H_31_N_4_O_6_S, 455.1959).

### In vitro folding

D-core streptavidin peptide and natural core streptavidin (Roche Diagnostics, 11,520,679) as lyophilized compounds were dissolved in 80 mM Tris–HCl, 1 mM EDTA, 6 M guanidine hydrochloride, pH 8.0. Each dissolved sample was heat-denatured at 85 °C for 45 min. The sample solution was diluted sixfold in the folding buffer (80 mM Tris–HCl, 1 mM EDTA, pH 8.0) at 25 °C for 30 min and re-diluted 100-fold in the same buffer at 4 °C for 18 h. Final solution condition of each step was shown in Table 2. Each diluted sample was concentrated 200-fold using a centrifugal filter unit (Merck Millipore) and kept at 4 °C for 12 h.

### Analytical SEC

SEC was run using a Hitachi HPLC system equipped with a Chromaster HPLC pump 5110, UV–VIS detector 5420, and autosampler 5210 (Hitachi High-Technologies). UV absorbance at 280 nm was analyzed using Chromaster System Manager version 1.1 (Hitachi High-Technologies). A SEC column (Superdex200 Increase 10/300, 10 mm φ x 300 mm, Cytiva) was equilibrated with arginine-based SEC eluent buffer (Arg-SEC Mobile Phase standard type, NACALAI TESQUE) at a flow rate of 0.8 mL/min at 25 °C for more than 2 h. A column was loaded with 10 µg (in 20–50 µL) of core streptavidin samples and protein concentrations of detected peaks at 280 nm were quantified using a standard peak area (10 µg) of reagent core streptavidin (Roche Diagnostics, 11520679).

### Far UV CD spectrum analysis

The measurement samples were prepared by dilution to 300 μg/mL in 20 mM phosphate buffer. CD spectra (Far UV region: 190–260 nm) were acquired at 25 °C using a CD spectrometer (JASCO, J-1500). The path length was set to 1 mm. The data pitch was set to 0.1 mm. The scanning speed was set to 100 nm/min, and the spectra were averaged from three scans. The spectral baseline was recorded using a 20 mM phosphate buffer. Each data point was baseline-subtracted.

### DSF analysis

DSF analysis was performed using the Uncle and Uncle Analysis software version 5.01 (Unchained Labs). The samples were diluted to 0.5 mg/mL with 20 mM phosphate buffer. The samples were subjected to a thermal ramp from 20 to 95 °C at a ramp rate of 1 °C/min.

### SPR analysis

#### Preparation of biotin labeled albumin

50 mg/ml of bovine serum albumin (2.8 ml; Proliant Biologicals, 68,700) was added to 10 mg/mL of L-biotin-AC5-OSu in DMF solution (163 µL) and the mixture was incubated at 35 °C for 1 h. The mixture was buffer-exchanged to 0.1 M phosphate buffer (pH 7.5) with PD-10 column (Cytiva). D-biotin labeled albumin was produced under the same conditions using D-biotin-AC5-OSu (Dojin Chemical).

#### Measurement

The Surface plasmon resonance was measured using a Biacore T200 instrument (Cytiva). Each prepared biotin labeled bovine serum albumin was immobilized on each flow cell of a CM5 sensor chip by the amine coupling method to a final targeted resonance unit of 400. Non-biotin labeled bovine serum albumin (Proliant Biologicals, 68,700) was immobilized on a reference flow cell under the same condition. Next, five concentrations (0.05, 0.15, 0.45, 1.35, and 4.05 nM) of D-core streptavidin in HBS-EP + (Cytiva) buffer were passed over the chip using a single cycle method. The sensorgram data were analyzed using Biacore T200 evaluation software version 3.1. K_D_ was calculated by fitting curve analysis with 1:1 binding kinetics and equilibrium analysis with steady-state affinity and 1:1 binding. Each analysis was performed in a single cycle using five concentrations (0.05, 0.15, 0.45, 1.35, and 4.05 nM).

### Immunoassay

#### Preparation

1. D-core streptavidin (1 μg/mL Phosphate buffered saline (PBS), 100 μL) was added to a 96-well ELISA plate (Thermo Fisher Scientific, 436,110) and incubated at 4 °C overnight. After washing three times with PBS, PBS buffer containing 2% bovine serum albumin (200 μL; Proliant Biologicals, 68,700) was added and shaken for 2 h at 25 °C with a rotation speed of 600 rpm. The natural core streptavidin immune assay plate was prepared under the same conditions using reagent core streptavidin (Roche Diagnostics, 11,520,679).
2. 5.1 mg/mL thyroid-stimulating hormone (TSH) antibody (25 μL, Kitayama Labs, T 2–194) was added to 1.3 µL of 16.7 mg/mL of L-biotin-AC5-OSu in DMSO solution and the mixture was incubated at 35 °C for 1 h. The mixture was equilibrated with 0.1 M phosphate buffer (pH 7.5) PD-10 (Cytiva), and 500 μL fractions were separated and the fractions absorbing at 280 nm wavelength were collected. D-biotin labeled TSH antibody was produced under the same conditions using D-biotin-AC 5-OSu (Dojin Chemical).

### Evaluation of the effect on the measurement system in the presence of biotin

1. In a 1.5 mL tube, as a sample, HISCL TSH calibrator C3 (Sysmex) 400 μL and HISCL TSH R1 reagent (Sysmex) was added to 400 μL, and the reaction was carried out at 37 °C The reaction was carried out under the condition of 37 °C and 600 rpm for 30 min. To evaluate the influence of D-biotin, 0 ng/mL, 1 ng/mL, 10 ng/mL or 100 ng/mL of D-biotin was added to the samples and reacted.
2. The streptavidin-immobilized plate was washed three times with HISCL wash solution (Sysmex), and 60 μL of the reacted solution was added to each well (1.5-mL. The plates were washed three times with HISCL wash solution, and 60 μL of the reacted solution was added to each well. The reaction was carried out at 37 °C and 600 rpm for 20 min. Biotin-labeled TSH antibody prepared above was added, and the reaction was carried out at 37 °C and 600 rpm for 30 min.
3. The plate was washed five times with HISCL wash solution, and 50 μL of HISCL R4 Reagent (Sysmex) and 100 µL HISCL R5 Reagent (Sysmex) were added, and the reaction was carried out at 37 °C and 600 rpm for 3 min. After the reaction, luminescence was detected using a microplate reader (Infinite 2000 PRO, TECAN). The streptavidin-conjugated plate and biotin-labeled TSH antibody after blocking were also measured in the same way.

### Statistics

All values in Fig. 3 are expressed as mean from duplicated analysis.

## Supplementary Information

Supplementary Information.

## Data Availability

The data that support this study are available from the corresponding author upon reasonable request.
