# Supplementary material for: Mirror-image streptavidin with specific binding to L-biotin, the unnatural enantiomer
Source: Sci Rep. 2022 Jun 10;12:9568. doi: 10.1038/s41598-022-13763-4 (PMC9187662; doi:10.1038/s41598-022-13763-4)
Supplement: Supplementary file 1 — Supplementary Information. [file 41598_2022_13763_MOESM1_ESM.pdf]

Supporting Information

Mirror-image streptavidin with specific binding to L-biotin, the unnatural enantiomer

Masatoshi Suganuma[1,*], Takuya Kubo[2], Kengo Ishiki[2], Kota Tanaka[3], Kouzou Suto[3], Daisuke Ejima[4], Masahiro Toyota[5], Kouhei Tsumoto[6,7], Toshiyuki Sato[2], and Youichi Nishikawa[2]

[1]Central Research Laboratories, Sysmex Corporation, Kawasaki, 210-0821, Japan

[2]Central Research Laboratories, Sysmex Corporation, Kobe, 651-2271, Japan

[3]Bio-Diagnostic Reagent Technology Center, Sysmex Corporation, Kobe, 651-2271, Japan

[4]Bio-Diagnostic Reagent Technology Center, Sysmex Corporation, Sayama, 350-1332, Japan

[5]Department of Chemistry, Graduate School of Science, Osaka Prefecture University, Sakai, 599-8231, Japan

[6]Institute of Medical Sciences, The University of Tokyo, Tokyo, 108-8639, Japan

[7]Department of Bioengineering, School of Engineering, The University of Tokyo, Tokyo, 113-8656, Japan

Supplemental methods

Chemical peptide synthesis
Abbreviations
Boc, *tert*-butoxycarbonyl; Boc_2O, di-*tert*-butyl decarbonate; *t*Bu, *tert*-butyl; CD, circular dichroism; DIC, *N*,*N'*-diisopropylcarbodiimide; DCM, dichloromethane; DIPEA, diisopropylethylamine; DMF, *N,N*-dimethylformamide; EDT, 1,2-ethanedithiol; equiv., equivalent; ESI MS, electrospray ionization mass spectrometry; Fmoc, 9-fluorenylmethyloxycarbonyl; Gdn, guanidinium; Hmb, 2-hydroxy-4-methoxybenzyl; HMPB, 4-(4-hydroxymethyl-3-methoxyphenoxy)butyryl; HPLC, high performance liquid chromatography; MESNA, sodium 2-mercaptoethane sulfonate; MPAA, 4-mercaptophenylacetic acid; MW, microwave; NMP, *N*-methyl-2-pyrrolidone; Oxyma Pure, ethyl cyano(hydroxyamino)acetate; Pbf, 2,2,4,6,7-pentamethyldihydrobenzofuran-5-sulfonyl; Pi, Phosphate; PyBOP, (benzotriazol-1-yloxy)tripyrrolidinophosphonium hexafluorophosphate; TCEP, tris(2-carboxyethyl)phosphine hydrochloride; TFA, trifluoroacetic acid; TFE, 2,2,2-trifluoroethanol; TIS, triisopropylsilane; Trt, trityl; UV, ultra-violet; v/v, volume per volume.

Materials and methods
Reagents and solvents
Fmoc-D-amino acid derivatives and Fmoc-Gly were purchased from Merck & Co., Inc. (NJ, USA). All other reagents and solvents were purchased from Wako Pure Chemical Industries, Ltd. (Osaka, Japan), Tokyo Chemical Industry Co., Ltd. (Tokyo, Japan), Nacalai Tesque, Inc. (Kyoto, Japan), Watanabe Chemical Industries, Ltd. (Hiroshima, Japan), Merck KGaA (Darmstadt, Germany) and Sigma-Aldrich Co. LLC. (MO, USA).

Characterization and purification
Preparative HPLC was carried out on a Hitachi LaChrom Elite HPLC system (Hitachi High-Tech Corp., Tokyo, Japan) with a YMS triart C_{18} (20 × 250 mm) or an Osaka Soda CAPCELL PAK C_1 SG300 (4.6 × 250 mm) at flow rate of 9.9 mL/min or 1.0 mL/min, respectively, using a binary mixture of A (0.1% TFA in H_2O) and B [CH_3CN/H_2O/TFA (v/v, 90/10/0.09)] eluents. Separation was performed using the described linear gradient and

detection at 220 nm. Analytical HPLC was performed on a Hitachi LaChrom Elite HPLC system with an Osaka Soda CAPCELL PAK C_1 SG300 (4.6 × 250 mm) using a binary mixture of A and B eluents. The analysis was performed using a linear gradient at a flow rate of 1.0 mL/min and detection at 220 nm. ESI MS experiments were conducted on a Synapt HDMS mass spectrometer (Waters, MA, USA).

Solid phase peptide synthesis

Automated peptide synthesis by Fmoc solid-phase peptide synthesis was carried out on a Liberty Blue automated MW peptide synthesizer (CEM Corp., NC, USA). The peptide chain was elongated using the standard Fmoc protocol of coupling with Fmoc-amino acid/DIC/Oxyma Pure/DIPEA (5.0/5.0/5.0/0.5 equiv.) in DMF (2 min, 90 ºC) except for the D-Asp-Gly sequence, which was incorporated using Fmoc-D-Asp(*t*Bu)-(Hmb)Gly (1.5 and 1.0 equiv., double coupling) with DIC/Oxyma Pure (1.5/1.5 equiv.) in NMP at room temperature by the manual procedure to avoid the aspartimide side reaction arising from the repetitive base treatment using 20% piperidine/DMF. Deprotection of N^{α}-Fmoc groups was performed using 20% piperidine/DMF (0.5 min, 90 ºC). During the peptide synthesis, all washings after couplings and deprotections were performed with DMF. The following side chain-protecting groups were employed: *t*Bu for D-Asp, D-Glu, D-Ser, D-Thr and D-Tyr, Boc for D-Lys and D-Trp, Pbf for D-Arg, Trt for D-Asn, D-Cys, D-Gln and D-His.

Synthesis of D-core streptavidin peptide

The D-form of core streptavidin peptide (D-core streptavidin peptide) consisting of D-amino acids and the achiral amino acid glycine was assembled from two segments (D-Ala^{1}-D-Thr^{59}) and (D-Cys^{60}-D-Ser^{127}) by native chemical ligation followed by desulfurization (Scheme S1).

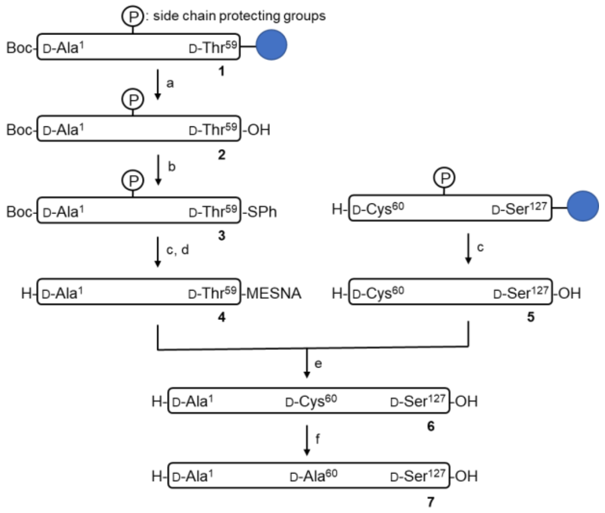

Figure S1. Synthetic route for D-core streptavidin peptide (1-127) **7**. Reagents and conditions: a, 1% TFA/DCM; b, PhSH, PyBOP/DIPEA; c, TFA/TIS/EDT/H_2O (v/v, 92.5/2.5/2.5, 3.0 mL); d, MESNA/Pi buffer (pH 7.0); e, MPAA, TCEP/Pi buffer (pH 7.1) containing 8M Gdn·HCl and 40 mM ascorbic acid; f, VA-044, TCEP, 2-methyl-2-propanthiol/Pi buffer (pH 7.1) containing 8M Gdn·HCl.

Synthesis of D-core streptavidin peptide (1-59)-thioester 4

<u>(i) Loading of Fmoc-D-Thr(*t*Bu) onto HMPB-ChemMatrix resin</u>

HMPB-ChemMatrix resin (Biotage, 0.82 g, 0.40 mmol) was placed in a peptide synthesis reactor to which DCM was added to cover the resin with three times the bed volume. The reaction vessel was shaken gently for 1 h. To the resulting resin was added a solution of Fmoc-D-Thr(*t*Bu) (0.80 g, 2.0 mmol), 1-(mesitylene-2-sulfonyl)-3-nitro-1*H*-1,2,4-triazole (0.60 g, 2.0 mmol) and 1-methylimidazole (0.16 mL, 2.0 mmol) in DCM (12 mL). The reaction mixture was agitated for 3 h at room temperature.

<u>(ii) Chain assembly of D-core streptavidin peptide (1-59)</u>

After washing the resin with DMF, Fmoc-Gly^{58} (5.0 equiv.), Fmoc-D-Ser(*t*Bu)^{57} (5.0 equiv.) and Fmoc-D-Asp(*t*Bu)^{56}-(Hmb)Gly^{55} (1.5 and 1.0 equiv., double coupling) were sequentially elongated onto a portion of the Fmoc-D-Thr(*t*Bu) loaded resin (0.10 mmol) by the manual procedure using the DIC/Oxyma Pure method. The

resulting pentapeptide resin was transferred to the MW-assisted automated synthesizer and peptide elongation was recommenced. During the peptide chain elongation reaction, the D-Asp^{24}-Gly^{25} sequence was manually incorporated using Fmoc-D-Asp(*t*Bu)-(Hmb)Gly as described above, followed by acetyl capping using acetic anhydride/Oxyma Pure/DIPEA (170 μL/5.3 mg/79 μL) in NMP (7.0 mL) at room temperature. After chain assembly was completed, the peptide resin was treated with 20% piperidine/DMF to remove the N-terminal Fmoc group and then washed with DMF and DCM successively.

(iii) Preparation of D-core streptavidin peptide (1-59)-thioester **4**

The peptide resin (0.10 mmol) obtained above was treated with a solution of Boc_2O (0.12 mL, 0.50 mmol) and DIPEA (87 μL, 0.50 mmol) in NMP (3.0 mL) at room temperature for 1 h. After the procedure to introduce the Boc group into the N-terminal amino group was repeated, the resin **1** was washed with NMP and DCM successively and then dried. The segment was detached from the resin by treatment with 1.0% TFA/DCM (3.0 mL) for 5 min to obtain it in the form of a fully protected peptide with a free α-carboxy group. The filtrate was collected in a vessel containing pyridine (0.6 mL). After this procedure was repeated 13 times, the resin was washed three times with TFE/DCM (v/v, 3/5) and the washings were combined with the filtrate. Solvents were removed under *vacuo* to give a residue, which was precipitated by adding water (40 mL) to give the protected peptide **2** (0.17 g, 20%). To the protected D-core streptavidin peptide (1-59) **2** (0.12 g, 14 μmol) in NMP (5.0 mL) was added thiophenol (42 μL, 0.40 mmol), PyBOP and DIPEA (12 μL, 68 μmol), and the reaction mixture was stirred at −15 ºC overnight. The reaction mixture was poured into ice-cold ether (40 mL) to afford a precipitate, which was washed with ether, collected by centrifugation (× 10000 rpm, 10 min) and dried (0.11 g). The crude protected peptide-thiophenylester thus obtained was treated with TFA/TIS/EDT/H_2O (v/v, 92.5/2.5/2.5, 3.0 mL) at room temperature for 3 h to remove all protecting groups. The product was precipitated by adding ice-cold ether (40 mL) to give the peptide-thiophenylester **3** (86 mg), which was subsequently converted into the peptide-thioester **4** by treating it with 0.2 M phosphate buffer (3.0 mL, pH 7.0) containing 8 M guanidium chloride and 0.2 M MESNA at room temperature for 1 h. The reaction mixture was purified on an Osaka Soda CAPCELL PAK C_1 SG300 column (4.6 × 250 mm) using a linear gradient of 17.5–21.5% B (flow rate, 1.0 mL/min; detection, 220 nm) in 12 min at 60 ºC to give the peptide-thioester **4** (6.6 mg, 7.9% calculated from **2**). ESI MS: *m/z* calcd for $C_{258}H_{393}N_{69}O_{95}S_2$ $[M+6H]^{6+}$ 1008.58, $[M+5H]^{5+}$ 1210.10, $[M+4H]^{4+}$ 1512.38, $[M+3H]^{3+}$ 2016.17; found 1008.47, 1209.96, 1512.21, 2015.98.

Synthesis of [D-Cys^{60}]-D-core streptavidin peptide (60-127) 5

The peptide segment **5** was synthesized from Fmoc-D-Ser(*t*Bu)-HMPB ChemMatrix resin (0.10 mmol) by employing the same procedure as described for D-core streptavidin peptide (1-59)-thioester **4** to yield the crude product (0.40 g), which was purified by preparative HPLC on a YMC triart C_{18} column (20 × 250 mm) using a linear gradient of 30–45% B (flow rate, 9.9 mL/min; detection, 220 nm) in 30 min at 60 ºC. Yield: 37 mg (5.0%). ESI MS: *m/z* calcd for $C_{329}H_{496}N_{94}O_{100}S$ $[M+8H]^{8+}$ 926.03, $[M+7H]^{7+}$ 1058.17, $[M+6H]^{6+}$ 1234.37, $[M+4H]^{4+}$ 1851.04; found 925.83, 1058.08, 1234.28, 1850.93.

Synthesis of D-core streptavidin peptide (1-127) *7*

(i) Preparation of [D-Cys^{60}]-D-core streptavidin peptide (1-127) **6**

The N-terminal unprotected peptide (1–59)-thioester **4** (6.0 mg, 0.93 μmol) and the C-terminal (D-Cys^{60}-127) peptide **5** (9.3 mg, 1.3 μmol) were solubilized in freshly degassed 0.2 M sodium phosphate buffer (pH 7.1, 0.55 mL) containing 8 M guanidium hydrochloride, 40 mM MPAA, 40 mM ascorbic acid and 40 mM TCEP. The reaction mixture was allowed to react under stirring for 24 h at room temperature. Next, the reaction mixture was treated with 0.2 M sodium phosphate buffer (pH 7.0, 0.55 mL) containing 8 M guanidium hydrochloride and 0.2 M MESNA for 1 h and then purified on an Osaka Soda CAPCELL PAK C_1 SG300 column (4.6 × 250 mm) using a linear gradient of 15–35% B (flow rate, 1.0 mL/min; detection, 220 nm) in 30 min at 60 ºC to give [D-Cys^{60}]-D-core streptavidin peptide (1-127) **6** (6.7 mg, 54% calculated from **4**). ESI MS: *m/z* calcd for $C_{585}H_{883}N_{163}O_{192}S$ $[M+17H]^{17+}$ 783.56, $[M+16H]^{16+}$ 832.47, $[M+15H]^{15+}$ 887.90, $[M+14H]^{14+}$ 951.25; found 783.50, 832.39, 887.94, 951.14.

Desulfurization to convert [D-Cys^{60}]-D-core streptavidin (1-127) **6** to D-core streptavidin (1-127) **7**

To a solution of [D-Cys^{60}]-D-core streptavidin (1-127) **6** (4.7 mg, 0.35 μmol) in degassed 0.2M sodium phosphate

buffer (0.70 mL, pH 7.1) containing 8 M guanidine hydrochloride and 0.25 M TCEP was added VA-044 (6.8 mg) and 2-methyl-2-propanthiol (40 µL, 0.38 µmol). The mixture was stirred at room temperature for 4 h and then diluted with 0.2 M sodium phosphate buffer (1.4 mL, pH 7.1) containing 8 M guanidium chloride and 0.2 M ascorbic acid. The resulting solution was partitioned with ether (1.7 mL) three times and the aqueous layer was directly subjected to HPLC purification on an Osaka Soda CAPCELL PAK C_1 SG300 column (4.6 × 250 mm) using a linear gradient of 15–45% B (flow rate, 1.0 mL/min; detection, 220 nm) in 30 min at 60 ºC to obtain D-core streptavidin (1-127) **7** (2.6 mg, 55%). ESI MS: *m/z* calcd for $C_{585}H_{883}N_{163}O_{192}$ $[M+17H]^{17+}$ 781.67, $[M+16H]^{16+}$ 830.47, $[M+15H]^{15+}$ 885.76, $[M+14H]^{14+}$ 948.96, $[M+13H]^{13+}$ 1021.87, $[M+12H]^{12+}$ 1106.94, $[M+11H]^{11+}$ 1207.48, $[M+10H]^{10+}$ 1328.13, $[M+9H]^{9+}$ 1475.59, $[M+8H]^{8+}$ 1659.91, $[M+7H]^{7+}$ 1896.89, $[M+6H]^{6+}$ 2212.88; found 781.63, 830.41, 885.76, 948.89, 1021.81, 1106.91, 1207.41, 1328.04, 1475.53, 1659.88, 1896.77, 2212.78.

ESI-MS for peptide

Peptide was dissolved in CH_3CN aqueous solution containing 0.1% TFA. The solution was injected at a flow rate of 20 µL/min into the ESI interface of a Synapt High-Definition Mass Spectrometer (Waters). The instrument parameters of ESI-MS were as follows: N_2 drying gas of 350 L/h and 150 °C and capillary voltages of 3.0 kV for positive-ion mode.

HPLC for peptide

HPLC was performed using a CAPCELL PAK C_1 SG300(4.6 x 250 mm, Osaka soda) at 60°C. Eluent A consisted water containing 0.1% TFA and eluent B consisted of 90% CH_3CN aqueous containing 0.09% TFA. The mobile phase gradient ranged from 15% to 45% (eluent B) for 30 min at a flow rate 1.0 mL/min. UV absorbance was measured at 220 nm.

Near UV CD spectrum analysis

The measurement samples were prepared by dilution to 1.4 mg/mL in 20 mM phosphate buffer. CD spectra (Near UV region: 250-300 nm) were acquired at 25 °C using a CD spectrometer (JASCO, J-1500). The path length was set to 1 mm. The data pitch was set to 0.5 mm. The scanning speed was set to 100 nm/min, and the spectra were averaged from three scans. The spectral baseline was recorded using a 20 mM phosphate buffer. Each data point was baseline-subtracted.

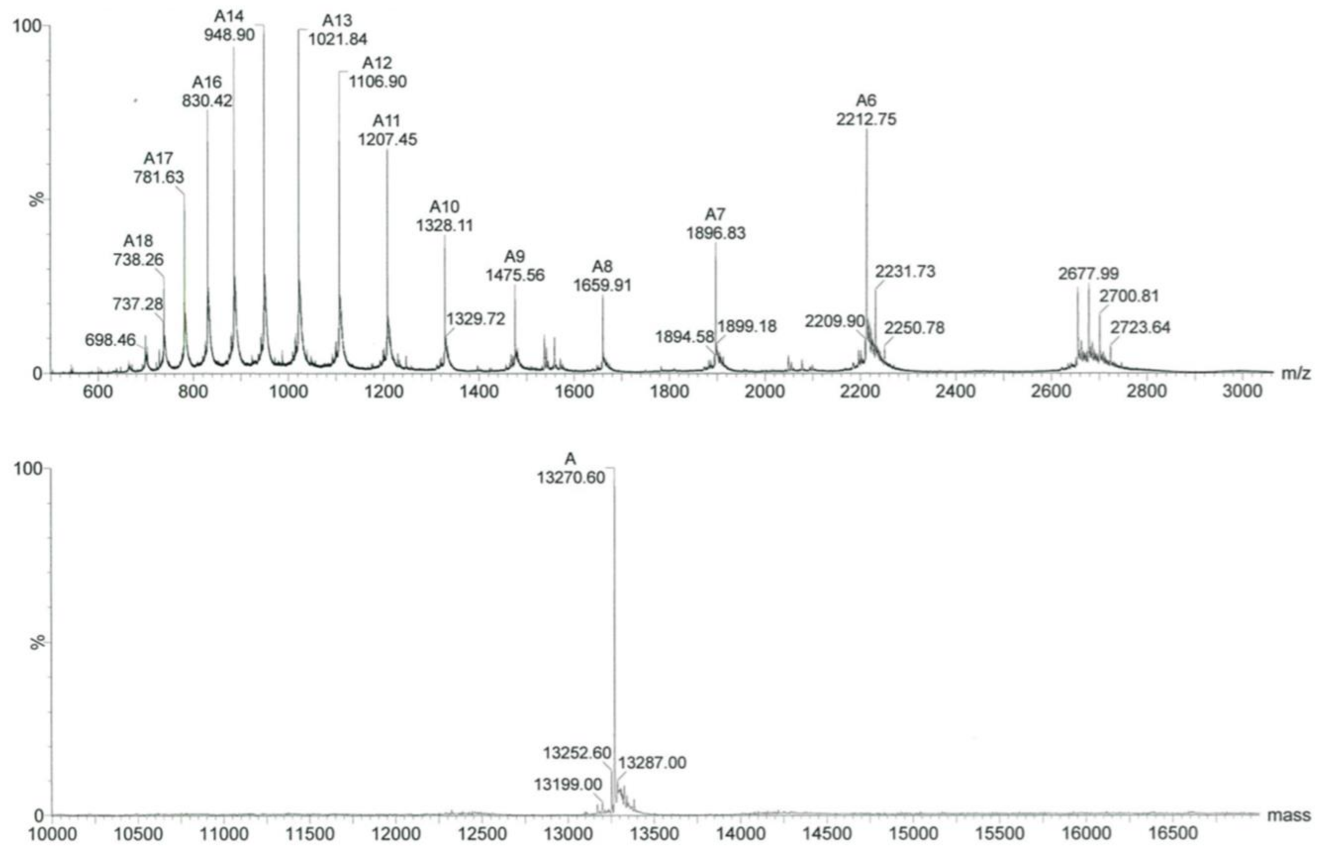

Figure S2. ESI-MS spectrum of D-core streptavidin peptide. Theoretical molecular weight of the peptide is 13271.4 g/mol.

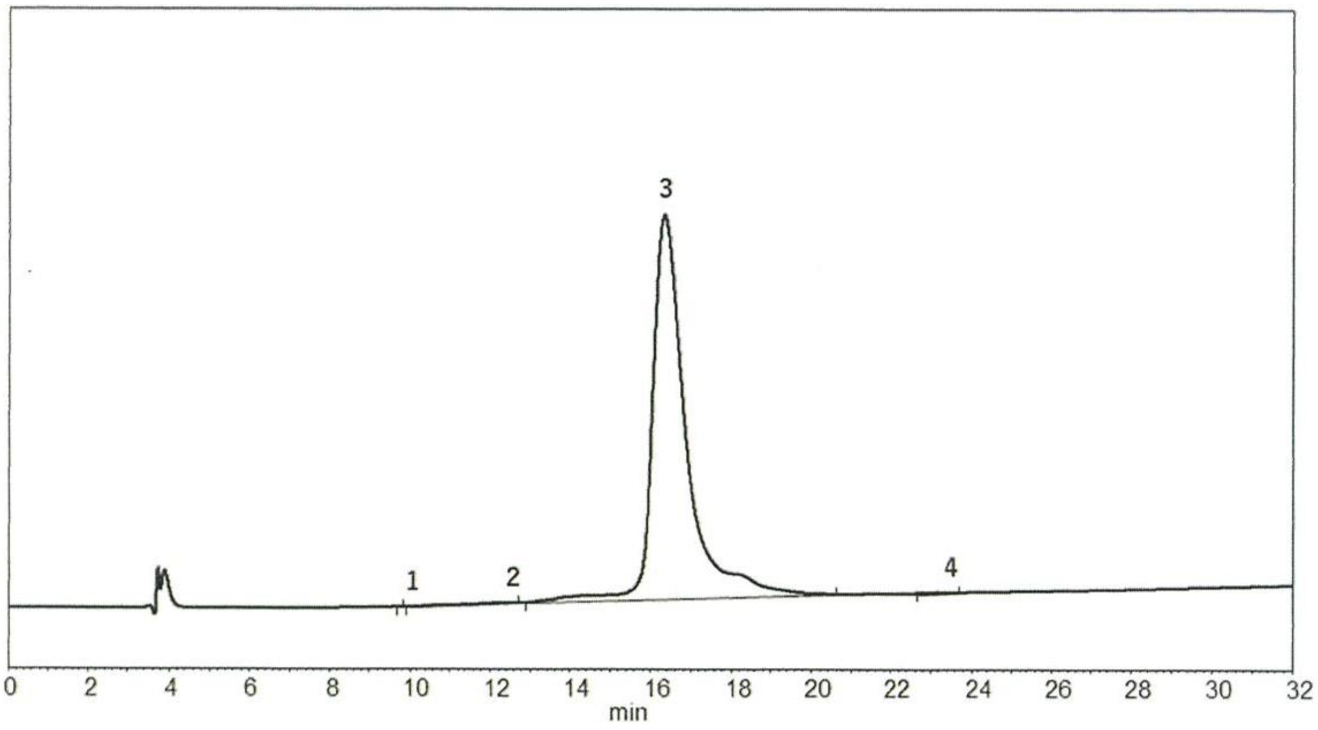

Figure S3. HPLC spectrum of D-core streptavidin peptide.

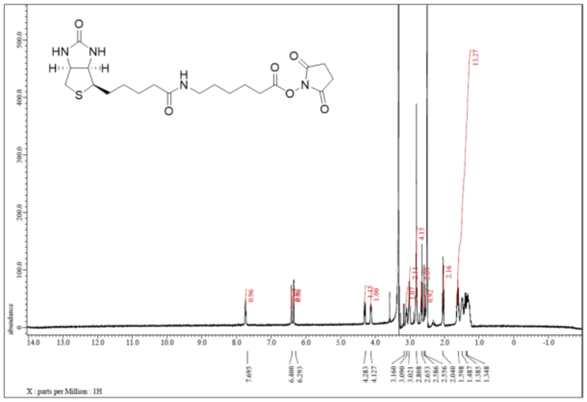

Figure S4. ^{1}H NMR spectrum of L-biotin-AC5-OSu **4** in DMSO-d_6.

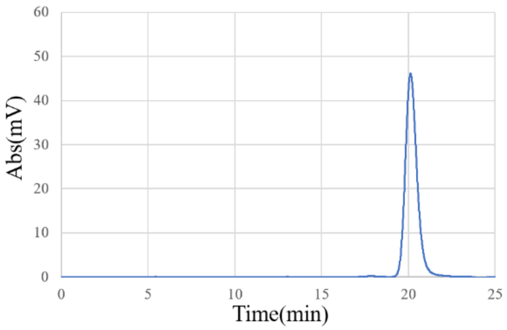

Figure S5. SEC of reagent natural core streptavidin tetramer (Roche Diagnostics, 11520679)

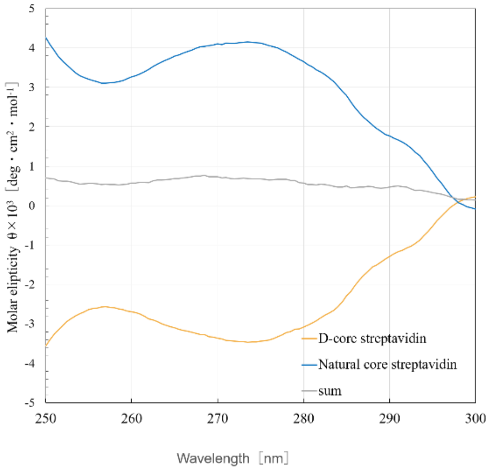

Figure S6. Near UV CD spectra for D- and natural core streptavidin

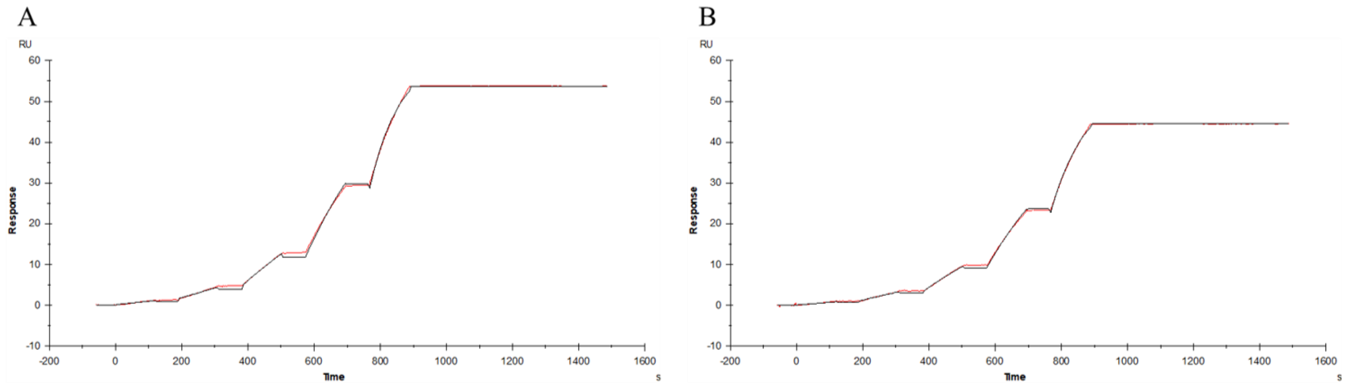

Figure S7. Fitting curve for fitting curve analysis. A: L-biotin and D-core streptavidin, B: D-biotin and natural core streptavidin

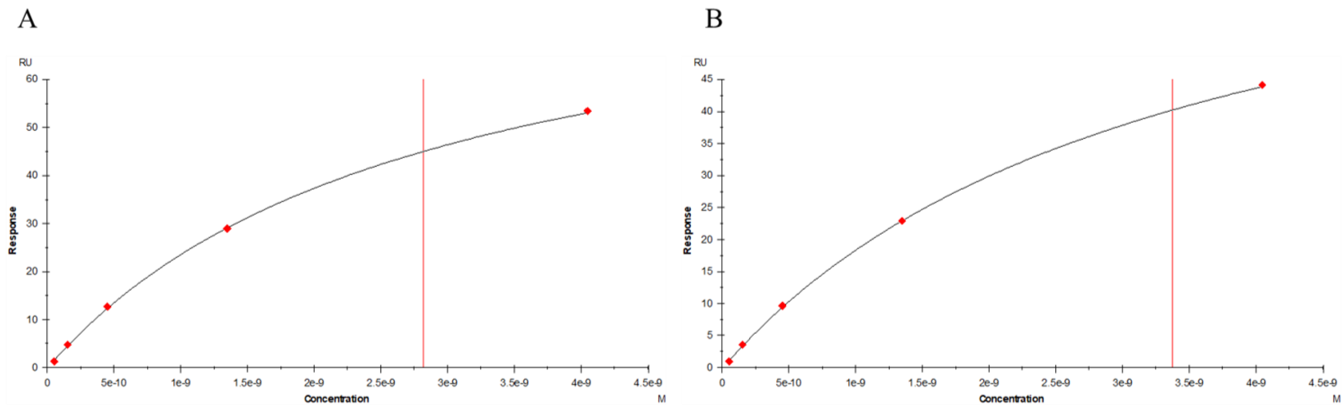

Figure S8. Steady-state affinity analysis. A: L-biotin and D-core streptavidin, B: D-biotin and natural core streptavidin
